# Improving accuracy of protein-protein interaction prediction by considering the converse problem for sequence representation

**DOI:** 10.1186/1471-2105-12-409

**Published:** 2011-10-24

**Authors:** Xianwen Ren, Yong-Cui Wang, Yong Wang, Xiang-Sun Zhang, Nai-Yang Deng

**Affiliations:** 1 State Key Laboratory for Molecular Virology and Genetic Engineering, Institute of Pathogen Biology, Chinese Academy Medical Sciences & Peking Union Medical College, Beijing, 100730, China; 2 College of Science, Chinese Agricultural University, Beijing, 100083, China; 3 Key Laboratory of Adaptation and Evolution of Plateau Biota, Northwest Institute of Plateau Biology, Chinese Academy of Sciences, Xining, 810001, China; 4 Academy of Mathematics and Systems Science, Chinese Academy of Sciences, Beijing, 100190, China

## Abstract

**Background:**

With the development of genome-sequencing technologies, protein sequences are readily obtained by translating the measured mRNAs. Therefore predicting protein-protein interactions from the sequences is of great demand. The reason lies in the fact that identifying protein-protein interactions is becoming a bottleneck for eventually understanding the functions of proteins, especially for those organisms barely characterized. Although a few methods have been proposed, the converse problem, if the features used extract sufficient and unbiased information from protein sequences, is almost untouched.

**Results:**

In this study, we interrogate this problem theoretically by an optimization scheme. Motivated by the theoretical investigation, we find novel encoding methods for both protein sequences and protein pairs. Our new methods exploit sufficiently the information of protein sequences and reduce artificial bias and computational cost. Thus, it significantly outperforms the available methods regarding sensitivity, specificity, precision, and recall with cross-validation evaluation and reaches ~80% and ~90% accuracy in *Escherichia coli *and *Saccharomyces cerevisiae *respectively. Our findings here hold important implication for other sequence-based prediction tasks because representation of biological sequence is always the first step in computational biology.

**Conclusions:**

By considering the converse problem, we propose new representation methods for both protein sequences and protein pairs. The results show that our method significantly improves the accuracy of protein-protein interaction predictions.

## Background

The concerted interactions of thousands of proteins in cells form the basis of most of the biological processes. Genome-wide identification of protein-protein interactions is important to understand the underlying mechanisms of many biological phenomena e.g. cell cycles, apoptosis, signal transduction, and pathogenesis of diseases. Recently, high-throughput experimental methodologies have been developed to screen the protein-protein interactions (PPIs) in a genome-wide way, e.g. yeast two-hybrid systems [1], mass spectrometry [2,3], and protein microarrays [4,5]. But these genome-wide studies are limited to a few of model organisms, for example, *Escherichia coli *[6], *Helicobacter pylori *[7], *Saccharomyces cerevisiae *[3,8,9], *Caenorhabditis elegans *[10], *Drosophila melanogaster *[11], and *Homo sapiens *[12,13]. These preliminary explorations provide valuable resources to study the model organisms [14]. More importantly, it allows us to learn the interacting rules from the available PPIs to construct a universal predictor for accelerating the mapping of whole interactomes of organisms, especially those species barely characterized.

To construct a universal predictor, we need to extract protein attributes that are crucial to PPIs predictions. Among the various attributes of proteins, the primary sequences are the most basic and the easiest to obtain because of the rapid development of genomic sequencing technologies. In addition, the primary sequences of proteins virtually specify their structures that provide the molecular basis for PPIs. So protein primary sequences hold the promise to contain virtually sufficient information to construct the most universal predicting method [15].

We know that almost all proteins are composed of twenty amino acids but different proteins have various lengths. Here the first challenge to construct a universal PPI predictor is that how to represent the various lengths of proteins by numerical vectors with the same dimension if vector-based computational methods are used. Even if the methods are not based on vectors, what features of the protein sequences are important to PPIs should be addressed first. So far, many methods have been proposed [15-20]. However the converse problem, that is, to what extent the protein sequences can be reconstructed based on their vector representations, is often untouched. Obviously, addressing this converse problem will facilitate the comparison of various representation schemes. Here, we develop an optimization model to evaluate theoretically the qualities of various representation schemes by considering the converse problems of protein representation as well as the computational costs.

Based on the key ingredients revealed by the optimization model, we suggest new coding methods for both protein sequences and protein pairs. Strict evaluations on datasets of *Escherichia coli *and *Saccharomyces cerevisiae *suggest that our new vector representation for protein sequences improves the prediction accuracy significantly while reducing greatly the computational complexity. The new vector representation of the protein pairs further improves the prediction accuracy and has excellent theoretical properties, i.e., symmetry, reversibility, and unbiasedness.

## Results

### Evaluating the converse problem of protein vector representations

We consider two theoretical aspects to evaluate various vector representations of protein sequences. One is to what extent the protein sequence information is extracted by the vectors. This can be evaluated by checking whether and how protein sequences can be constructed conversely from the vectors. The other is how the vector dimension increases as the information extracted. Because of the curse of dimensionality, representations with low vector dimension are appreciated in real applications. These criteria can be summarized as the following optimization model:

(1) mindim(V)

s.t.

(2) V=f(S)

(3) S=g(V)

where *S *is a set of protein sequences, *V *is the vector representation of *S *generated by the mapping *f *and *g *is the inverse function of *f*. dim(*V*) means the dimension of *V *.

Based on the evaluation model, we compared the available *k*-mer based (denoted by K) [15,16,20] and segmentation based (denoted by P) [21] vector representations. *k*-mer based representation counts the number of each *k*-mer appearing in protein sequences, so the vector dimension is 20^*k*^, increasing exponentially as *k*. When *k *is large enough (often much larger than three), protein sequences can be re-constructed uniquely from the corresponding vectors by seeking an Eulerian trail in a network constructed by the relationships of *k*-mers. Segmentation-based methods divide a protein sequence into *p *pieces and then count the number of each amino acid appearing in each piece. So the resultant vector dimension is 20*p. When *p *is equal to the length of protein sequence, the protein sequence can be reconstructed easily by filling amino acids in each segment because there is only one amino acid in each segment. When *p *is less than the length of protein sequence, some sequence information is lost and the protein sequence cannot be reconstructed exactly.

**Figure 1:**
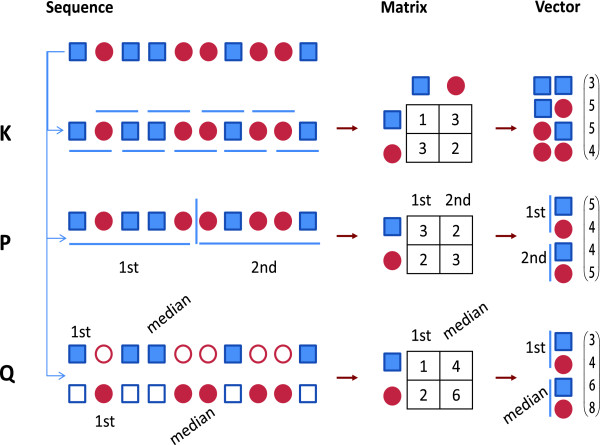
**A toy example to illustrate the encoding schemes for protein sequences**. Given a toy sequence of two letters, k-mer based methods, denoted by K, count the number of each k-mer in the sequence. Here k = 2. The counting process is represented as a matrix in which the rows represent the first letter of 2-mers and the columns represent the second letter of 2-mers. The dimension of the resultant vector is 2^2 ^= 4. If k = 3, the dimension will be 2^3 ^= 8. For real protein sequences, the dimension will be 20^3 ^= 8, 000. Segmentation based methods, denoted by P, divide the sequence evenly into p pieces first and then count the number of each letter in each piece. Here p = 2. The dimension of the resultant vector is 2*2 = 4. If p = 3, the dimension will be 2*3 = 6. For real protein sequence, the dimension will be 20*3 = 60. Quantile based methods, denoted by Q, record the positions of q quantiles of instead of the number of each letter. Here q = 2 and the first and the median positions of each letter are recorded.

Inspired by the reversibility and low-dimension requirements of the evaluation model and the fact that protein sequences are "sequences", we propose a new vector representation scheme by recording the positions (denoted by Q). Q treats the positions of each type of amino acids as a distribution and records the *q *quantile positions of each type of amino acids. A toy example is illustrated in Figure 1. The dimension of the resultant vectors of Q method is 20*q, increasing linearly as *q*. Because position information is complementary to the amino acid or *k*-mer counts, super representation schemes, for example, QP and KQP, can be constructed. For instance, QP divides a protein sequence to *p *pieces and then counts the number and records the *q *quantile positions of each type of amino acids in each piece, resulting a 20*(1+*q*)*p vector. KQP divides a protein sequence to *p *pieces and then counts the number and records the *q *quantile positions of each *k*-mer in each piece, resulting a 20^k^*(1+*q*)*p vector. A detailed comparison of these representing methods is summarized in Table 1. In summary, we find that QP vectors are expected to extract more information with low dimension and the follow-up experimental results suggest the advantage of this method.

**Table 1:** Features of various representation schemes of protein sequence according to our evaluation model

	K	Q	P	QP	KQP
f	Counts	Positions	Counts	Counts and positions	Counts and positions
g	Eulerian trails, reversible	Simple filling, reversible	Simple filling, reversible	Simple filling, reversible	Eulerian trails and filling, reversible
dim(*V*)	20^k^	20*q	20*p	20*(1+q)*p	20^k^*(1+q)*p

K: k-mer based methods; P: segmentation based methods; Q: our quantile-position based methods; QP: combination of Q and P; KQP: combination of K, Q and P. f: mapping from sequences to vectors; g: mapping from vectors to sequences; dim(*V*): dimension of vector V.

### The converse problem of vector representation of protein pairs

To predict PPIs, we need further encode protein pairs into a single vector. The reversibility requirement also applies to the vector representation of protein pairs. Here, symmetry is the first condition that must be satisfied. Protein-protein interaction is widely believed to be symmetric interaction in biology [22], i.e., protein A interacting with protein B has the same meaning with the fact that protein B interacts protein A. For example, protein-protein interaction networks are always treated as undirected graphs [23] because proteins bind together and have no explicit direction. In this sense protein-protein interactions are mutual, therefore the representation of protein pairs should be naturally symmetric. Otherwise the predicting result for AB may be inconsistent with that of BA. Available symmetry solutions for protein pairs either work on vector level, e.g., abs(*ν*_*A*_-*ν*_*B*_) [19], or work on kernel level, e.g., [15,24], but do not consider the reversibility. Here we propose a new solution based on the symmetry of sum and multiplication operations (denoted by SM). By applying arithmetical and geometric average operations additionally, a second refined scheme is given (denoted by AG). For SM, given the vector representations of Protein A (*ν*_*A*_) and Protein B (*ν*_*B*_), we construct two new vectors: one is *ν*_*A*_+*ν*_*B *_and the other is *ν*_A_*ν_*B*_, in which * means the corresponding elements multiplication. Then the two symmetric vectors are concatenated into one vector. For AG, the arithmetical average of ν_*A *_and *ν*_*B *_(denoted by *ν*_*AM*_) and the geometric average of *ν*_*A *_and *ν*_*B *_(denoted by *ν*_*GM*_) are calculated. That is, the i-th dimensional element of *ν*_*AM *_and *ν*_*GM *_are given by the following formulations:

(4) $$v_{AM}^{i}=\frac{1}{2}\left(v_{A}^{i}+v_{B}^{i}\right)$$

(5) $$v_{GM}^{i}=\sqrt{v_{A}^{i}*v_{B}^{i}}$$

When *ν*_*AM *_and *ν*_*GM *_are calculated, the symmetric representation of protein pair (A, B) will be the concatenation of *ν*_*AM *_and *ν*_*GM*_. AG has three important properties: 1) The resultant vector is symmetric regarding to protein pairs (A, B) and (B, A) because of the commutative laws of addition and multiplication; 2)For each dimension *i*, $v_{A}^{i}$ and $v_{B}^{i}$ can be reversely constructed from $v_{AM}^{i}$ and $v_{GM}^{i}$ by solving Equations (4) and (5); 3)Each dimension of the symmetric representation is of the same scale as the original vectors *ν*_*A *_and *ν*_*B *_because of the average operations, without artificial noise introduced. These three properties facilitate the extraction of information in the protein vectors and are beneficial to learning the rules underlying PPIs (see results for more detailed discussions).

### Overview of performances of various methods

**Figure 2:**
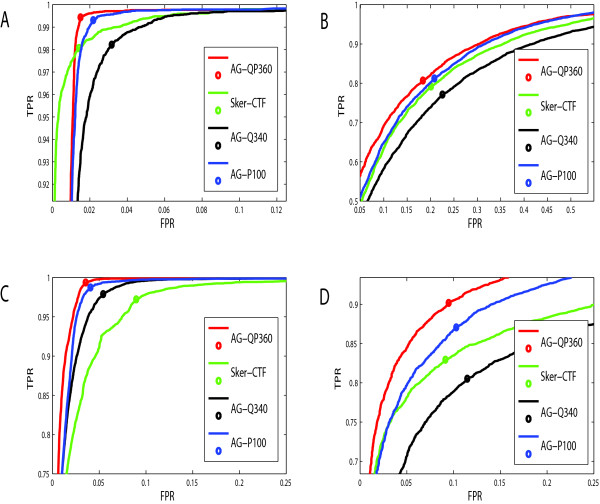
**The ROC curves of four available predicting methods on *Escherichia coli *and *Saccharomyces cerevisiae *datasets**. A, ROC curves on *Escherichia coli *data with negative samples constructed by sub-cellular information; B, ROC curves on *Escherichia coli *data with negative samples sampled randomly from the complementary network; C, ROC curves on *Saccharomyces cerevisiae *data with negative samples constructed by subcellular information; D, ROC curves on *Saccharomyces cerevisiae *data with negative samples sampled randomly from the complementary network.

**Figure 3:**
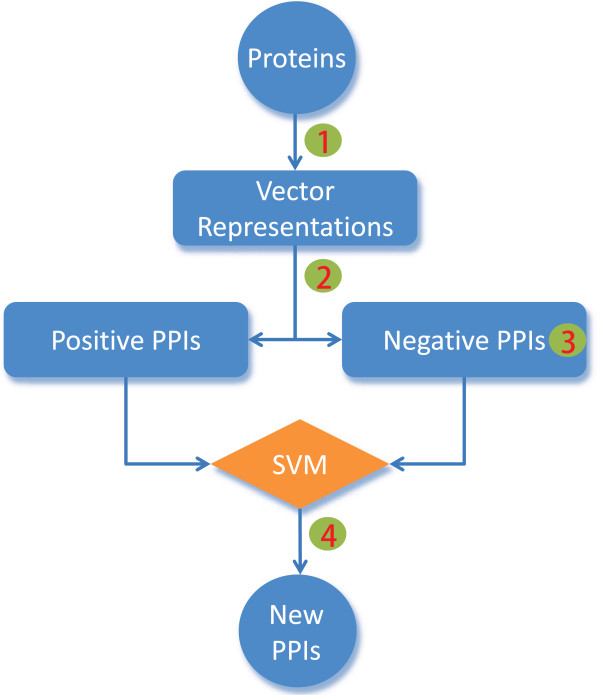
**Flow chart for applying SVMs to predict PPIs from sequences**. Four issues must be addressed. First, protein sequences must be represented by vectors. Second, the vector representation of protein pairs must be symmetric. Third, a set of non-interacting protein pairs (negative PPIs) should be provided because SVMs are supervised learning algorithms. Fourth, a proper kernel will facilitate the nonlinear prediction. The focus of this paper is on the first and second issue. Community standard procedures are adopted to address the third and fourth issues in this paper.

We first compared our new proposals to two published methods (a *k*-mer-based method proposed by Shen et al. [15] and a segmentation-based method proposed by Luo et al. [21] on the model organisms *Escherichia coli *and *Saccharomyces cerevisiae *with two types of negative samples (Figure 2). The Receiver Operating Characteristic (ROC) curves show that our approach outperforms the other two available methods (Figure 3), suggesting that it may extract more information which is essential to PPIs. The advantage of our approach is due to both the new vector representation of protein sequences and the novel symmetric representation of protein pairs. Strict evaluation of them is as follows.

### Comparison of symmetric representation methods of protein pairs

As we mentioned, the representation of protein pairs should be symmetric. Otherwise the predicting result for AB may be inconsistent with that of BA. Here we compared four symmetric representing schemes. One scheme is |*ν*_*A*_*-ν*_*B*_|, denoted by dist. It is on the vector level and used in [19]. The other is proposed by Shen *et al*. and is on the kernel level [15]. The conjoint triad method proposed by Shen *et al*. is used for all the four schemes as the vector representation of protein sequences to guarantee the fairness of the comparison. The conjoint triad method is a variant of k-mer method that classifies twenty amino acids into seven families [15]. These four solutions are denoted by AG-CTF (A: arithmetical, G: geometric, CTF: conjoint triad features), dist-CTF (dist: distance), sker-CTF (sker: S kernel, the name of the kernel proposed by Shen et al), and SM-CTF (S: sum, M: multiplication), respectively. The comparison is conducted on *Escherichia coli *and *Saccharomyces cerevisiae *data sets with two types of negative samples. "Benchmark negatives" means that the negative samples are from the sub-cellular localization information. "Random negatives" means that the negative samples are sampled randomly from the complementary graph.

The comparison results are illustrated in Table 2. It can be seen that the AUC (the area under the ROC curve) value of dist-CTF is the least. This is because it ignores much information contained in the original vectors when constructing the symmetric vector representations. The other three solutions are comparable with a little difference regarding to AUC values. On *Escherichia coli *dataset with benchmark negative samples, sker-CTF achieves the highest AUC (0.998). AG-CTF reaches 0.996 and SM-CTF reaches 0.988. On the other three comparisons, AG-CTF always reaches the highest AUC values. And AG-CTF is better than SM-CTF because it solves the scale problem. Regarding the other indices, e.g. accuracy, sensitivity, specificity, and precision, AG-CTF also outperforms the other solutions. AG-CTF considers adequately the converse problem and solves the scale question, so its good performance is expected. Because it is based on the vector level, it is easy to track the physical meanings and the computation is efficient. The extremely high AUC values on the benchmark negative data sets are due to the bias incorporated during the construction of negative samples, which has been pointed out previously [25].

**Table 2:** The performance of four symmetric representing schemes for protein pairs

Organism	Methods	Benchmark negatives					Random negatives				
		
		AUC	Acc	Sn	Sp	Pre	AUC	Acc	Sn	Sp	Pre
*E. coli*	AG-CTF	0.996	**0.968**	**0.997**	**0.941**	**0.893**	**0.886**	**0.797**	**0.794**	**0.799**	**0.798**
	
	SM-CTF	0.988	0.948	0.985	0.929	0.879	0.876	0.788	0.785	0.789	0.789
	
	Sker-CTF	**0.998**	0.947	0.995	0.940	0.839	0.880	0.795	0.791	0.798	0.797
	
	Dist-CTF	0.955	0.892	0.842	0.899	0.786	0.769	0.702	0.704	0.699	0.701

*S. cerevisiae*	AG-CTF	**0.991**	**0.968**	**0.991**	**0.959**	**0.786**	**0.948**	**0.880**	**0.879**	**0.928**	**0.919**
	
	SM-CTF	0.990	0.964	0.984	0.958	0.766	0.939	0.868	0.837	0.899	0.893
	
	Sker-CTF	0.985	0.909	0.978	0.900	0.564	0.929	0.867	0.818	0.919	0.911
	
	Dist-CTF	0.946	0.891	0.826	0.900	0.523	0.849	0.788	0.764	0.799	0.792

Cutoff for each method was set according to the maximal F-measure statistic which is a community-standard procedure. Acc: accuracy; Sn: sensitivity; Sp: Specificity; Pre: precision.

### Comparison of vector representations of protein sequences

The above comparison reveals that the symmetry solution based on the arithmetical and geometric averages perform best. In this subsection, we choose to fix this strategy in the comparison of various vector representation schemes of protein sequences. In this way we can eliminate the differences introduced by different symmetric representations and make results rigorous. In total, four vector representation schemes of protein sequences are compared. They are: 1) conjoint triad features proposed by Shen et al. [15], denoted by AG-CTF; 2) segmentation-based method with *p *= 5[21], denoted by AG-P100; 3) position based method with *q *= 17, denoted by AG-Q340; and 4) the combination of segmentation and position with *p *= 3, *q *= 5, denoted by AG-QP360. *q *is set to seventeen for AG-Q340 because the resultant vectors have the almost same dimension compared to AG-CTF and AG-QP360. *p *= 3 and *q *= 5 for AG-QP360 is because of the same reason. We choose *p *= 5 for AG-P100 because it is a representative of this class of methods and reaches the best AUC value in cross-validation.

The comparison is illustrated in Table 3. On the benchmark negative data sets, these four representations achieve similar AUC values on both *Escherichia coli *and *Saccharomyces cerevisiae *data sets. On the *Escherichia coli *benchmark negative data set, AUC of AG-CTF reaches the highest 0.996. AG-QP360 and AG-P100 reach 0.994, which are a little bit smaller. AG-Q340 has the least AUC 0.989. On the yeast benchmark negative data set, AG-QP360 has the highest AUC 0.993 while AG-CTF, AG-P100, and AG-Q340 have AUCs 0.991, 0.991, and 0.989, respectively. Regarding the other indices including accuracy, sensitivity, specificity, and precision, AG-QP360 outperforms the other methods.

**Table 3:** The performance of four vector representing schemes for protein sequences

Organism	Methods	Benchmark negatives					Random negatives				
		
		AUC	Acc	Sn	Sp	Pre	AUC	Acc	Sn	Sp	Pre
*E. coli*	AG-QP360	0.994	**0.982**	**0.996**	**0.982**	**0.894**	**0.899**	**0.811**	**0.821**	**0.802**	**0.804**
	
	AG-CTF	**0.996**	0.968	0.987	0.940	0.889	0.886	0.797	0.794	0.799	0.798
	
	AG-P100	0.994	0.965	0.989	0.979	0.889	0.889	0.799	0.798	0.799	0.799
	
	AG-Q340	0.989	0.964	0.987	0.959	0.807	0.854	0.771	0.743	0.789	0.787

*S. cerevisiae*	AG-QP360	**0.993**	**0.968**	**0.998**	**0.969**	**0.786**	**0.960**	**0.902**	**0.887**	**0.929**	**0.917**
	
	AG-CTF	0.991	0.964	0.986	0.960	0.767	0.948	0.880	0.879	0.927	0.909
	
	AG-P100	0.991	0.963	0.985	0.959	0.765	0.947	0.849	0.798	0.899	0.889
	
	AG-Q340	0.989	0.945	0.982	0.939	0.684	0.902	0.844	0.788	0.898	0.877

Cutoff for each method was set according to the maximal F-measure statistic. Acc: accuracy; Sn: sensitivity; Sp: Specificity; Pre: precision.

Because of the bias in the benchmark negative data sets, each method can achieve very high AUC values but may limit its discriminating capacity. The negative samples sampled randomly from the complementary graphs are assumed to be unbiased so they may provide more discrimination power [25]. On the *Escherichia coli *random negative data set, AG-QP360 gets the highest AUC, 0.899, which is higher than that of AGP100 by one percent. AG-CTF has the third highest AUC (0.886) and AUC of AG-Q340 is the least (0.854). AG-QP360 also has the highest accuracy, sensitivity, specificity, and precision. On the *Saccharomyces cerevisiae *random negative data set, AG-QP360 still shows outperforming performances.

We also compared the performances of AG-QP360 and AG-CTF on the third type of negative samples to highlight the benefits of linearly scalable vector representations including segmentation based, position-based, and their combination (Table 4). Given a true protein sequence, uShuffle can generate artificial protein sequences that have the same composition of *k*-mers with the true sequence [26]. These artificial proteins have been used as negative samples in the previous studies to predict PPIs [27]. Here we construct three negative datasets of this type by reserving the composition of 1-mers, 2-mers and 3-mers, respectively. AG-QP360 performs well on all the three data sets but AG-CTF only performs well on the 1-mers and 2-mers datasets. On the 3-mers negative data set, AG-CTF loses its discriminative capacity because the conjoint triad features are in nature based on 3-mers. To get the discriminative power, k must increase to 4 or more but the vector dimensions will increase exponentially, aggravating greatly the computational burden and the dimensionality curse. Compared with that, the linearly scalable vector representations can handle this issue easily.

**Table 4:** AUC values of AG-QP360 and AG-CTF on the artificial negative data sets

Organism	Method	1-mer	2-mer	3-mer
*E. coli*	AG-QP360	0.966	0.932	0.914
	
	AG-CTF	0.957	0.936	-

*S. cerevisiae*	AG-QP360	0.969	0.931	0.918
	
	AG-CTF	0.956	0.936	-

-: The method has no power to discriminate positive PPIs from this type of negative PPIs.

Comparisons on human PPIs data were also implemented strictly (see SI Table 1, 2 and 3). The results on random negative samples and three types of shuffled negative samples all support the superiority of the new vector representations for both protein sequences and protein pairs.

## Discussion and conclusion

Predicting PPIs only from the sequence information is an important and challenging problem in the post-genomic era. We note that most current computational methods are trying to encode protein sequences with various lengths into vector with the same dimension. So the first inevitable question for successful prediction is how to encode protein sequences effectively and efficiently in vector spaces. Previous studies propose various encoding methods but seldom consider the converse problem. In this study, we propose an evaluation model and analyze the available *k*-mer based methods and segmentation based methods by investigating the converse problem, and suggest that when *k *or *p *is large enough, a protein sequence corresponds to a unique vector. But the dimension of the resultant vectors increases exponentially for *k*-mers based methods and linearly for segmentation based methods. And *k*-mer based methods emphasize extracting the local information while segmentation based methods emphasize the global information.

Viewing the protein sequences as distributions of amino acids, we propose a new dimension-linearly-increasing vector representation scheme for protein sequences by recording the positions of *q *quantiles of each type of amino acids. It can serve as an independent encoding method and can also combine with segmentation based methods to form super methods, whose dimension increases still linearly with the scaling parameters *p *and *q*. Experiments on *Escherichia coli *and *Saccharomyces cerevisiae *datasets with various types of negative samples suggest the outperforming power of the proposed super methods. Comparisons on the artificial negative samples further highlight the superiority of linearly scalable methods.

Applying the reversibility requirement on the symmetric vector representation of protein pairs results in a simple and reversible solution that is comparable to or even outperforms the available complicated kernels. Because it is based on the vector level, it is separated from the kernels and facilitates designing specific kernels to catch the nature of PPIs in the future.

Considering adequately the converse problem and seeking optimal representations has both theoretical and computational significance. It may theoretically point out the advantages and drawbacks of available methods and provide insights into how to improve the current methods. Furthermore, we only investigate the dictionary based encoding methods in this study. Physiochemical properties based methods are not investigated but they are ready to be incorporated into our framework as the additional information other than sequence. We think the information holds their potential to unravel the physical and chemical principles underlying the interactions.

Obviously, there are a lot of other unsolved questions in predicting computationally PPIs. For example, proteins interact with each other through certain domains or building blocks rather than the global sequences. Which parts are essential to protein interactions and how to computationally identify them need more deep investigations. The second limitation of sequence-based predictions is how to predict remote PPIs across organisms. Currently the predicting accuracy of remote PPIs is much lower than the intra-organism predictions. We note that the current domain databases may provide a few clues. However, their bias and incompleteness, especially information loss, should also be considered adequately. Another question is that the gold standard negative samples of PPIs are missing. Various methods have been proposed to construct the negative samples to highlight the patterns embedding in the positive data sets. But artificial biases are also introduced. How to construct unbiased negative samples is a big issue and still in argument currently.

## Methods

### The benchmark data and predicting methods

Numerically, we evaluate the vector encoding methods and our improvements with support vector machines (SVM) on *Escherichia coli *and *Saccharomyces cerevisiae *PPIs datasets. SVMs are one type of the state-of-the-art supervised machine learning methods and have been used extensively in various disciplines including bioinformatics. Here we use SVMs to evaluate various representation schemes. Details of SVMs can be found in refs [28]. Other learning methods are also qualified to do evaluation but the selection of learning methods is not the focus of this paper. Four general issues must be addressed when applying SVMs to predicting PPIs (Figure 3). First, protein sequences must be represented by vectors. Second, the vector representation of protein pairs must be symmetric. Third, gold-standard negative data (a set of non-interaction protein pairs) should be provided because SVMs are supervised learning algorithms. Fourth, a proper kernel will facilitate the prediction greatly. Since the focus of this paper is only related to the first and the second issues, community standard solutions are adopted to address the third and fourth issues in this paper. Specifically, we use three types of negative samples which have been widely used in the previous studies for predicting PPIs. The first type is constructed manually based on the sub-cellular localization of proteins, which assumes that proteins with different sub-cellular localizations are not prone to interact. The second type is sampled randomly from the complementary graph of the PPIs network, which assumes the sparseness of the PPIs network. The third type is constructed by disturbing randomly the amino acid sequences of interacting protein pairs while conserving the composition of amino acids or *k*-mers by uShuffle [26]. Yu *et al*. propose a fourth method for constructing the negative PPI samples by imposing the degree distribution of the positive PPI set to the negative PPIs [29]. They propose an excellent question what roles the special network structures of PPIs networks play in PPIs prediction. However, we argue that the requirement of the same degree distribution of the positive and negative PPI sets is not reasonable (the complementary graph of a PPIs network cannot be of the same degree distribution as the PPIs network). So this type of negative PPIs was not suitable for evaluating the performances of PPIs prediction from sequences. Despite that the PPIs networks are assumed to be sparse, we select randomly the same number of the negative samples to do the evaluation. If more negative samples are included, the unknown true PPIs may also be included as negative samples. The positive and the first type of negative data of PPIs are from [19] that were manually curated for quality. We use soft-margin SVM to resolve the remaining errors in the data. All the evaluations are conducted by five-fold cross-validations. Gaussian kernels are adopted for the fourth issue and the parameters are tuned by a grid search.

The protein sequences are from the RefSeq database of NCBI. PPIs involving proteins whose sequences are not available are filtered. Finally, 6,962 positive interactions are included in the cross-validation experiments for *Escherichia coli *and 6,635 positive interactions are included in the cross-validation experiments for *Saccharomyces cerevisiae*. The numbers of negative samples are the same as the number of positive samples for balance. Human PPIs were downloaded from the Human Protein Reference Database (HPRD) on Dec. 21st, 2009 [30].

Protein sequences are converted into vectors by four schemes (CTF, P100, Q340, and QP360). CTF classifies the twenty amino acids into seven classes and then applies *k*-mer based method with *k *= 3. The details can be found in [15]. P100 divides a protein sequence into five pieces first and then counts the number of each type of amino acid. Q340 records seventeen quantile positions for each type of amino acid. QP360 first divides a protein sequence into three pieces, then counts the number of each type of amino acid and records five quantile positions for each type of amino acid in each piece. Each protein sequence is normalized according to its length. That is, the elements of the resultant vector are divided by the length of the protein sequence. The symmetric representations of protein pairs include four methods (dist, Sker, SM and AG). Given *ν*_*A *_and *ν*_*B*_, dist generates the symmetric vector by abs (*ν*_*A*_*-ν*_*B*_). Sker calculates the kernel matrix according to the S kernel defined in [15]. SM creates the symmetric vector by concatenating *ν*_*A*_*+ν*_*B *_and *ν*_A_*ν_*B *_in which * means the multiplication of the corresponding elements. AG gets the symmetric representation according to (4) and (5). libsvm 2.88 [31] is used to implement the algorithms of support vector machines on a PC machine with Intel Core 2 Due CPU 2.83 Hz. The Gaussian kernel is applied. The parameters are tuned by a grid search method and the optimal ones are (C = 10, γ = 0.025) for CTF methods and (C = 10, γ = 0.0125) for other methods. All the evaluations are conducted in five-fold cross-validations.

## Authors' contributions

XR proposed the idea for this work. XR and YCW designed the predictive methods and the experiments, prepared the experiments and wrote the paper. YW analyzed the results and revised the paper. XSZ and NYD participated in developing the methods and revised the article. All authors read and approved the final manuscript.
